# The Kumagai Method Utilizing the Pigeon Bottle Feeder with a Long Nipple: A Descriptive Study for the Development of Feeding Techniques for Children with Cleft Lip and/or Palate

**DOI:** 10.3390/children11030365

**Published:** 2024-03-19

**Authors:** Shingo Ueki, Yukari Kumagai, Yumi Hirai, Eri Nagatomo, Shoko Miyauchi, Takuro Inoue, Qi An, Junko Miyata

**Affiliations:** 1 Department of Health Sciences, Faculty of Medical Sciences, Kyushu University, Fukuoka 812-8582, Japan; 2 Department of Nursing, Osaka University Dental Hospital, Suita 565-0871, Japan; 3 Department of Advanced Information Technology, Faculty of Information Science and Electrical Engineering, Kyushu University, Fukuoka 819-0395, Japan; 4 Department of Informatics, Graduate School of Information Science and Electrical Engineering, Kyushu University, Fukuoka 819-0395, Japan; 5 Department of Human and Engineered Environmental Studies, Graduate School of Frontier Sciences, The University of Tokyo, Kashiwa 277-8563, Japan

**Keywords:** child, feeding, cleft lip, cleft palate, descriptive study, long nipple

## Abstract

We aimed to identify the steps involved in the Kumagai method—an experimental nursing procedure to feed children with cleft lip and/or palate, using a feeder with a long nipple. We conducted a descriptive study, enrolling five specialist nurses who have mastered the Kumagai method. Their approaches were examined using structured interviews. Moreover, the participants were asked to perform the sequence of actions involved in this method while describing each step. Therefore, we were able to explore the Kumagai method in depth and step-by-step, including the following aspects: correct infant posture; correct feeding bottle holding position; nipple insertion into the child’s mouth; and feeding process initiation, maintenance, and termination. Each step comprises several clinically relevant aspects aimed at encouraging the infant to suck with a closed mouth and stimulating chokubo-zui, i.e., simulation of the natural tongue movement during breastfeeding in children without a cleft palate. In conclusion, when performed correctly, the Kumagai method improves feeding efficiency in children with cleft lip and/or palate. Feeders with long nipples are rarely used in clinical practice; the Kumagai method might popularize their use, thereby improving the management of feeding practices for children with cleft lip and/or palate.

## 1. Introduction

For infants, milk is essential for their growth and constitutes their primary source of nutrition. Infants drink the presented milk automatically because of the sucking reflex. When sucking, milk extraction is caused by the movement of the tongue, which undulates like a generating peristaltic wave [1], and the occurrence of a vacuum when the tongue is in the lowest position [2]. However, some infants cannot create the necessary negative pressure in the oral cavity owing to conditions such as cleft lip and/or palate (CLP).

CLP is a congenital anomaly with a prevalence of 9.92 per 10,000 births worldwide [3], the highest prevalence among oral and maxillofacial malformations. According to commonly used surgical guidelines for CLP, attainment of “a weight of 5 kg” indicates the optimal timing of surgical cleft lip repair [4]. In infants with cleft lip alone or accompanied by a small cleft palate, the child can achieve negative pressure, and feeding is often successful without needing specific interventions [5]. However, many children with CLP have feeding difficulties [6] because the cleft impedes the generation of negative oral pressure [7]. In particular, children with Robin sequence exhibit CLP, micrognathia, glossoptosis, and upper airway obstruction, which are associated with severe feeding difficulties and poor weight gain [7]. Such children cannot be expected to gain weight promptly [8], and this may delay surgery [9]. 

**Figure 1:**
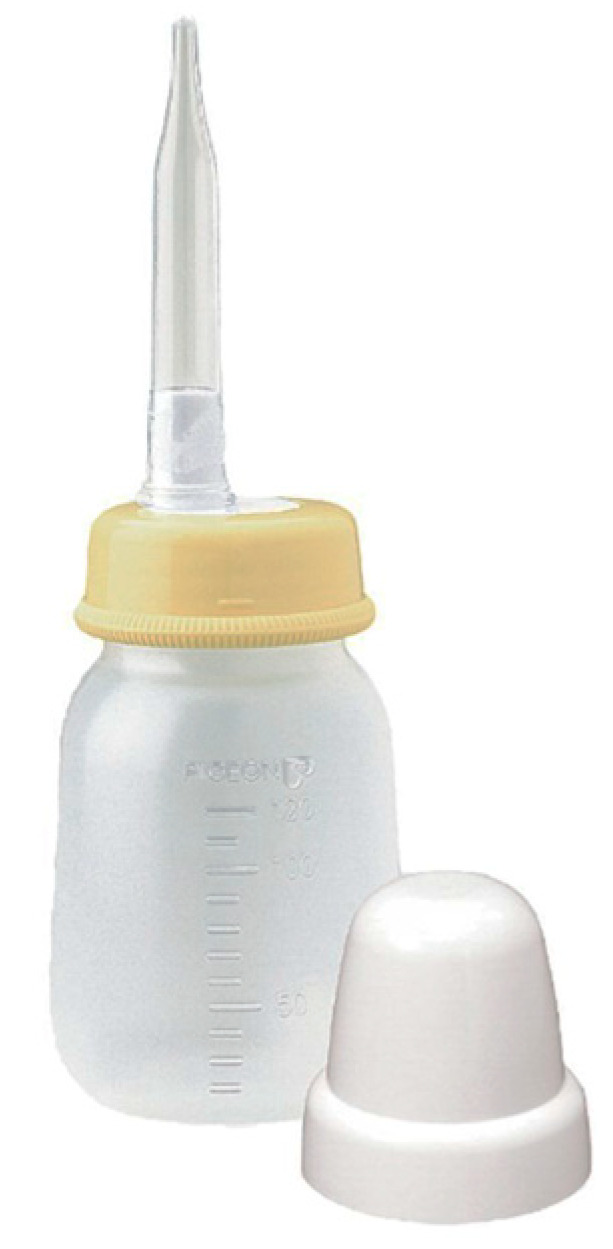
Pigeon bottle feeder with a long nipple.

For children with CLP who have feeding difficulties, many parents use bottle feeding exclusively or bottle feeding in combination with breastfeeding [10,11,12,13]. Some companies produce bottle nipples for children with CLP [14]. Table 1 presents the characteristics of feeding tools commonly used for children with CLP. The Pigeon Cleft Palate Soft Bottle and Medela Special Needs Feeder—which comprise a one-way valve and a squeezable bottle—are among the commonly used bottles for children with CLP [15]. With these bottles, it is possible to feed the infant by compressing the nipple or bottle without the child having to generate negative intraoral pressure [15]. However, these bottles have relatively large nipple diameters and are considerably more expensive than the standard feeding bottles [16]. The Pigeon bottle feeder with a long, soft, and thin nipple (hereafter referred to as Long Nipple) has a special shape and is less expensive than other nipples (Figure 1). This feeder is available for purchase in many countries, and in the current year (2024) it costs as little as approximately 4 or 5 US dollars. In clinical practice, it provides good milk dosing control, even for small doses; however, the optimal method for its use has not yet been established.

Bottle feeding methods specific to children with CLP have been described in several studies [7,15,17,18,19], including the following aspects: preparation before feeding (i.e., awaking by stimulation, improving the child’s mouth movement, or sealing the cleft with a finger), feeding time (approximately 20–30 min), feeding position (fairly upright), and feeding maintenance (squeezing the bottle every three to four sucks or stopping feeding two or three times to allow for burping). Although these techniques may be effective, the particular feeding bottle in which they demonstrate efficacy has not been clarified. Moreover, these techniques may not work in the case of the Long Nipple, which is conspicuously different in shape from other feeding equipment. Since each child with CLP has unique feeding difficulties, it is necessary to develop a range of feeding techniques for each feeding bottle that can be adapted to meet each child’s individual needs. Having various methods to choose from enables healthcare providers to better address the specific challenges presented by each infant. However, no studies have identified the optimal feeding methods specific to the Long Nipple for children with CLP. Consequently, the focus of this paper is to elucidate the feeding methods employed by CLP nursing specialists when using the Long Nipple for children with CLP.

## 2. Materials and Methods

### 2.1. Design

In the present study, we used a descriptive research design. 

### 2.2. Study Setting

The study was conducted at a specialized dental hospital in urban Japan affiliated with a university. The hospital’s CLP center has performed about 400 surgeries so far and supports 100 cases with feeding difficulties annually. Ms. Kumagai, the nursing department director, developed the Kumagai method after years of experience, taking advantage of the Long Nipple’s characteristics to optimize feeding for children with CLP, thus reducing physical strain and maximizing feeding volume.

### 2.3. Participants

The inclusion criteria for participation were nurses affiliated with the above dental hospital and certified as Long Nipple specialists. Among these, only five nurses had fully mastered the Kumagai method. Consequently, the study included these five nurses, including Ms. Kumagai. No exclusion criteria were established for participation.

### 2.4. Data Collection

Data collection started in September 2022. The participants were asked the following questions: “What kind of child requires the use of a Long Nipple?”, “What position do you assume when feeding?”, “How do you hold the bottle/nipple?”, “How do you insert the nipple?”, “How do you maintain feeding after inserting the nipple?”, and “How do you remove the nipple?”. The participants were asked to perform the correct and incorrect movements involved in milk feeding using the baby doll while verbally explaining the movements that corresponded to each question. These sessions were videorecorded.

### 2.5. Data Analysis

A content analysis [20] was performed for qualitative data analysis. First, we transcribed the recorded audio and then added a documented movement in the video. The qualitative data were read repeatedly to get acquainted with the source material. The feeding techniques were clearly separated into correct and incorrect movements and coded individually. The incorrect movement codes were used to clarify the meaning of the correct movement. The correct movement codes were categorized and subcategorized according to their semantic content. The categories were then organized into the temporal flow of the procedure, and a draft was created. The drafts were shown to the study participants to check for errors in their techniques. Finally, we asked Ms. Kumagai to check if there were any codes or categories that deviated from the Kumagai method and completed the dataset after adding and revising the relevant information.

### 2.6. Rigor Enhancement

To enhance the rigor of this study, we applied the principles of trustworthiness for qualitative data analysis [21]. The researcher who conducted the interviews specializes in pediatric nursing and has experience in nursing care for children with CLP in a pediatric unit but was unfamiliar with the Kumagai method. For confirmability, the researcher adopted neutrality and minimized preconceived assumptions by acknowledging its lack of knowledge on the matter and assuming a learner’s attitude toward the study participants. This data collection approach allowed us to interpret the participants’ voices more objectively.

To ensure data credibility, verbatim interview transcripts were provided to each participant to ensure data accuracy and completeness. Additionally, meetings were held between the researcher and study participants, including Ms. Kumagai, to discuss the results of the data analysis. Tables showing the process of coding and categorizing based on the entire raw dataset were shared with the authors involved with the data analysis process. Through three rounds of revision for credibility, a consensus was reached among all participants about the accurate description of the Kumagai method based on the generated categories and codes, and the appropriateness of the category names. Minutes of the meetings and the revision process were recorded and documented. An auditor was appointed at all times during the data collection and analysis for traceability to ensure dependability. For transferability, we provided a comprehensive report on the characteristics of the study sample, the data collection methods, and the interpretation process.

### 2.7. Ethical Considerations

The researcher (SU) explained the study outline to the participants based on the consent form and obtained their written consent. The consent form described the study purpose and methods, data storage, research funding, and conflicts of interest. Contact details were also provided. Additionally, it was also explained that participation was free and voluntary, and that withdrawing from the study would have elicited no negative consequences.

## 3. Results

### 3.1. Characteristics of Participants

The five nurses who participated in this study had 18–36 years of nursing experience. The average interview time was 40.1 min (range: 27.07–56.02).

The data obtained were analyzed and summarized into six themes: goals of the Kumagai method, basic posture, bottle handling, nipple insertion, feeding maintenance, and bottle removal. 

### 3.2. Goals of the Kumagai Method

The Kumagai method has two goals: “encouraging self-sucking with a closed mouth” and “being chokubo-zui” (in case of cleft lip, not palate). The word “chokubo-zui” translates from Japanese as “sucking as breastfeeding”. It is defined as the form and motion of the child’s mouth and tongue while sucking: the child’s tongue forms a U-shape, the entire tongue adheres to the nipple, and negative pressure is created through the waving motion of the tongue.

When the nurses feed a child using the Long Nipple, they aim to maximize the child’s primitive sucking movements and allow the pediatric patient to drink like a healthy child. They referred to a specific form of tongue-supported sucking as “chokubo-zui”. They understood that their adherence to the Kumagai method leads a child without a cleft palate to be this “chokubo-zui” and increases the child’s feeding volume. They also mentioned that drinking milk with the child’s mouth closed was a sign that they did not dislike the nipple, even in children with a cleft palate.

*I defined the word “chokubo-zui” to describe that the child’s tongue is shaped like a U and that the nipple is caught by the entire tongue to create negative pressure for drinking. A healthy child drinks breast milk in a similar way; the tongue is shaped like a U, setting the nipple into a wave-like motion when drinking. A child without a cleft palate does this when drinking. It is the correct way to drink, or maybe I should say that it is similar to breastfeeding, which increases the child’s level of satisfaction. When the “chokubo-zui” begins to appear, the child’s sucking becomes more stable, and the necessary amount of milk can be obtained. Therefore, the Kumagai method aims to induce this “chokubo-zui”.*

*(Participant D)*

*I believe that only when all the Kumagai method I have described are performed carefully, will the child accept the nipple and be able to drink by self-sucking with his lips tightly closed. Drinking milk with the mouth closed indicates that the child has accepted the nipple.*

*(Participant C)*

### 3.3. Basic Posture

The two categories of basic positions for holding the child while feeding were “cuddling as if pulling the child toward oneself” and “holding the child straight in a slightly upright position” (Table 2a). The participants held the child close to their body to limit the range of motion of the child’s body so that the child could concentrate on feeding. The raw data representative of this category are shown in Table 3, which also shows the coding and categorization process.

The participants also mentioned that seating the child so that their body is slightly upright prevents aspiration and reduces the child’s discomfort.

*The child’s body should be held as straight as possible without directing the chin too much upward or downward. Ideally, the angle of the child’s body should be in a slightly upright position. The reason for this approach is that the nipple must be inserted into the mouth so that the tongue and nipple are parallel, and if the child’s body leans back too much, the nipple must be tilted down to keep it parallel with the tongue. If you do this, milk will automatically drip down, and the child will have a negative perception of the nipple. The important part is to keep the nipple and the tongue parallel, which is why I hold the child at this angle.*

*(Participant B)*

### 3.4. How to Hold the Bottle

**Figure 2:**
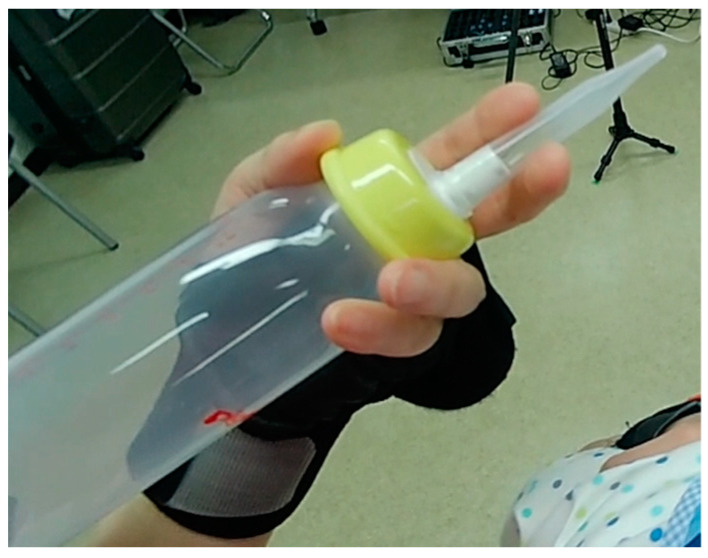
How to hold the bottle.

Unlike most bottles, while holding a Long Nipple bottle, it must be ensured that the nipple can be pinched with two fingers (Table 2b); Figure 2 shows how to hold the bottle accordingly.

The Long Nipple comprises four components: a bottle, bottle cap, holder, and the Long Nipple itself. The nipple is connected to the convex portion of the holder, which is off-centered. Consequently, to ensure that the last of the milk in the bottle flows out, the convex portion of the holder must be positioned on the bottom side when the bottle is tilted. When holding the bottle with the remaining three fingers, it is important to ensure that the nipple hole does not face downward in the child’s mouth. The tip of the Long Nipple is cross-cut, with a hole in one section. This design causes the milk to flow in a non-linear direction. Therefore, it is important to consider the direction of the milk flow when attaching the nipple for feeding.

*When I use this Long Nipple for a child with swallowing problems, I take care of the direction of the nipple hole. When attaching the nipple to the holder, make sure the nipple hole does not point downward. Ideally, the nipple hole should be on the right or left side, but it is good enough if the hole position is not downward. The milk squirts onto the side of the nipple where the hole is. If the hole is facing down, the milk streams directly toward the child’s throat, which is dangerous. If the child can drink without choking, you do not need to worry much about the direction of the hole.*

*(Participant A)*

The participants regulate the amount of milk provided by pinching the Long Nipple between two fingers. They are also careful about the pinching position to prevent the nipple from moving inside the child’s mouth.

*This (pinching) position should be in the center of the nipple where the nipple is thicker. The reason for holding at this location is because it produces the least amount of shaking. The nipple should be inserted in a taut, pinned position by maintaining the nipple like a stick so that it does not bend. The finger (holding the nipple) should be as relaxed as possible. By slightly curling the finger, unnecessary force from the finger is prevented, and the tongue movement is better transmitted. By keeping the fingers relaxed, the child’s lips and mouth will be free from unnecessary force.*

*(Participant E)*

Regarding how to hold the bottle, three participants said, “*I support the bottle from its sides, like parallel to the arm, rather than from underneath or the top*”. Ms. Kumagai, however, said that this aspect was not an absolute requirement; therefore, it was omitted from the ultimate description of the Kumagai method reported herein.

### 3.5. Nipple Insertion into the Mouth

Three steps comprise the nipple insertion into the mouth (Table 4a). First, the bottle must be moved horizontally until the nipple touches the lips.

*Bring the nipple horizontally to the child’s lips. If the nipple is tilted toward the tongue, milk will flow.*

*(Participant B)*

Subsequently, the nipple is inserted into the mouth, onto the tongue, in a sliding motion. The wrist, not the elbow, controls the angle of insertion.

*First, gently place the nipple against the lower lip. Once the mouth is open, insert the nipple while tilting the nipple so that it is centered on the tongue to ensure contact of the nipple with the lip and the tongue. Placing the nipple on the lower lip, then inserting the nipple along the surface of the tongue will trigger the sucking reflex and smoothly start the sucking.*

*(Participant E)*

*The angles of the nipple are important, and how to achieve the angles with the arm holding the bottle is also important. Instead of raising the elbow to set the insertion angle, the angle should be set with the wrist only. This action makes it easier for us to manage the angle and facilitates feeding.*

*(Participant A)*

In the oral cavity, the nipple was positioned to maximize the utilization of the movement of the tongue manipulating the nipple. It was positioned at the midline and center of the tongue. They used the term “just point”, a Japanese-English word meant to accurately target the specific depth of the nipple insertion. During insertion, the fingers must support the nipple at all times to prevent it from bending.

*Generally, if you place this nipple at the center of the tongue, it will lead to a waving motion of the child’s tongue. Additionally, the child’s tongue wraps around the nipple in a U-shape, and the position of the nipple in the oral cavity becomes more stable. So you should place the nipple straight at the center of the tongue.*

*(Participant E)*

*The just point, we have said, is the depth of insertion of the nipple into the mouth; the nipple is inserted 2 or 3 millimeters beyond the border where the nipple thickens. Insertion of the nipple beyond the just point induces the vomiting reflex. Moreover, when the child raises the tongue during sucking, the nipple may bend in the mouth and touch the palate, making the child reluctant to continue. If the insertion is too shallow, only a very small amount of milk is released when sucking. The tongue may also feel the sharp tip of the nipple, making the child more reluctant to proceed.*

*(Participant D)*

*The nipple should remain straight, not bent or flexed; when the two fingers pinch the nipple, each finger should pinch with the same force, and the fingers should always stay in contact with the nipple even when the pressure is released.*

*(Participant D)*

**Figure 3:**
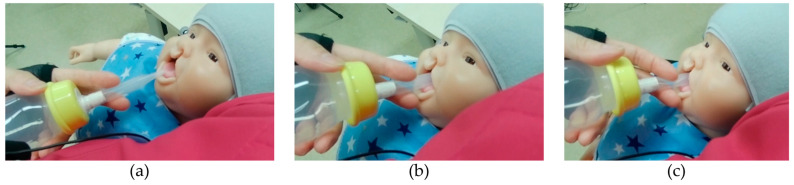
Process of nipple insertion into the mouth. (**a**) The nipple is lightly placed against the lower lip; (**b**) the nipple is placed into the mouth so that it adheres to the lower lip and the tongue; (**c**) the milk is pushed out by pinching the nipple between the index and middle fingers.

After inserting the nipple, the participants would immediately pinch the nipple between the fingers to push the milk out. This sequence of actions, i.e., from touching the lip to pinching the nipple, must occur within approximately 2 s to minimize the child’s resistance to feeding. This process is shown in Figure 3.

*Place the nipple on the lower lip (tap), put it in the mouth (swim), and pinch the nipple to push the milk out (push). The time of this “tap, swim, push” sequence is approximately 2 seconds. If you are too slow to insert the nipple, you will miss the time when the child desires to drink.*

*(Participant B)*

### 3.6. Maintaining Feeding

Proper placement of the nipple in the infant’s mouth is essential, as it affects milk production during feeding (Table 4b). To facilitate the child’s feeding, pinching the nipple should only be done while the child is actively sucking, not when inhaling air. It is important to observe the infant’s breathing and adjust the amount of milk accordingly by pinching the nipple; this ensures proper feeding while avoiding choking or aspiration.

*When the mouth is not closed, the child might be somewhat uncomfortable, so I try again to insert the nipple correctly.*

*(Participant B)*

*Always watch the child’s breathing when you push the nipple. We observe at what interval the child breathes and assess how much milk should be released continuously. We should control the amount of milk released according to the child’s breathing.*

*(Participant C)*

The amount of milk released by a single suction is estimated from the amount of air bubbles that flow into the nipple. The amount of milk considered suitable corresponds to approximately 1–2 cm of visible air bubbles in the nipple. The appropriate time for the infant to finish drinking the target amount of milk is approximately 15 min.

*I need to adjust the feeding speed until the child accepts the Long Nipple. The child will not be satisfied if the feeding is over in 5 min; meanwhile, sloppy drinking over 30 min will tire the mother.*

*(Participant D)*

### 3.7. End of Feeding

The participants are not careless when pulling out the nipple after feeding finishes. They are concerned about the Long Nipple movement to minimize child discomfort, even while pulling it out (Table 4c).

*It is poor practice to let the nipple bounce or pull it out against the upper lip while removing. If this is done before the child accepts the nipple, the child will feel discomfort. If the nipple is bent, milk will flow out. Therefore, if the tip of the nipple hits the mouth while removing the nipple, milk will enter the mouth, and the child will gag. If the nipple is filled with milk, milk will not flow out unless the feeding angle is changed. If the angle is changed or the nipple is bent, milk will come out unintentionally and may splash on the child’s face or enter the mouth, making the child feel uncomfortable.*

*(Participant D)*

## 4. Discussion

The Long Nipple utilized in this study has a unique shape and function, and improper usage by attempting to employ it as other feeding bottles may result in unintentional milk spillage and discomfort to the child. The Kumagai method, which makes the most use of the potential of the Long Nipple, is clearly different from approaches used for other feeding bottles, as discussed below.

The Long Nipple has two distinguishing characteristics compared with ordinary feeding bottles: the nipple is long, narrow, and made of soft material; and milk spills out when the bottle is tilted or when pressure is applied to the nipple [22]. The Medela Special Needs Feeder—which has a similar function to that of the Long Nipple—is a feeding bottle with a mechanism in which milk is pushed out when the nipple is pinched between the fingers. The material of the Medela nipple is relatively hard and requires pressure generated by the thumb and index fingers. By contrast, the Long Nipple is very soft; thus, the pressure generated by the index and middle fingers is sufficient for milk to flow out. Although it is possible to use the thumb, the elbow must be raised to tilt the bottle to push the milk out, and the arm tires when feeding this way for a long time. Pinching the Long Nipple with the index and middle fingers is more comfortable, while angling the bottle with the wrist.

As a method for pushing milk out of a Long Nipple, the company website states that the amount of milk can be adjusted by squeezing the bottle, which is made of soft polypropylene [23]. However, this method makes it difficult to fine-tune the amount of milk released. Children with CLP are prone to aspiration and otitis media [24]; therefore, the amount of milk the child can swallow must be finely adjusted. Moreover, supporting the nipple using the fingers was needed to prevent unexpected nipple bending. Therefore, this method of holding both the bottle and nipple seemed to be optimal.

The challenge with using the Long Nipple is the potential for milk to spill out if the bottle was tilted or if even the slightest pressure was applied to the nipple. Therefore, it was necessary to move the Long Nipple horizontally when bringing it to the child’s mouth. Additionally, it was recommended to pinch the nipple near its center using the fingers; the push should be performed with the same force, and the fingers should be kept on the nipple even when not pinching. These methods prevented the nipple from bending in the mouth, as well as unintentional milk spillages. The basic posture of “cuddling as if pulling the child toward oneself” was intended to restrict the child’s body movement, thereby preventing the nipple from moving when the infant moved unexpectedly, which also prevented unintentional milk spillage.

A slightly upright feeding posture had been recommended, whose purpose was to prevent milk aspiration [14]. However, in the Kumagai method, another purpose of this posture is to keep the Long Nipple horizontal while being parallel to the surface of the tongue as much as possible and to fit it into the shape of the tongue. The Kumagai method aims to promote feeding with a closed mouth and facilitate “chokubo-zui”, a technique in which the infant firmly supports the nipple with the tongue and controls the flow of milk at its own pace. To achieve these goals, it is essential that the nipple fits snugly against the shape of the infant’s tongue and that proper placement of the nipple and cuddling posture are maintained throughout the feeding process.

Like premature infants who may struggle with coordinating the processes of sucking, swallowing, and breathing [25], children with CLP must synchronize these three elements during feeding to prevent milk from flowing back into the nose [10]. In the present study, the participating specialists pushed milk out of the nipple while watching the timing of the child’s sucking and breathing, which was a measure to prevent accidental milk aspiration in children with CLP who have feeding difficulties. To comply with this timing, unintended milk spills must be prevented.

In the Kumagai method, some techniques were included to help the child accept and prefer the Long Nipple. One of the potentially essential steps for successful feeding is the insertion of the nipple in a sliding motion, applying a slight pressure on the nipple to push the milk out, which encourages the natural sucking reflex of the infant. The “tap, swim, push” time frame of approximately 2 s to prevent missing the child’s desire to drink at the right moment and the method of adjusting the pinching pressure according to the child’s sucking ability were strategies adopted to increase the child’s feeding satisfaction. To prevent the child from feeling uncomfortable with the nipple, the participants suggested keeping the nipple straight while feeding. Furthermore, maintaining the nipple at a constantly straight angle prevented unintended milk spillages and allowed the children to drink at their own pace; these techniques also helped the child accept the Long Nipple. Three nutritional goals for children with CLP have been defined [26]: to maintain nutritional status, to find feeding methods that are as close to normal as possible, and to identify feeding methods that maximize sucking stimulation. These elements are in the child’s best interest and promote oral motor development [26]. The Kumagai method is in line with these goals.

### 4.1. Strengths and Limitations of the Work

This study’s main strength lies in identifying the specific techniques for handling a uniquely shaped Long Nipple to address feeding difficulties in children with CLP. The method outlined in the study involves a set of precautions and considerations that differ significantly from those required for other feeding bottles. The Kumagai method, based on a thorough understanding of the Long Nipple’s function, harnesses its unique benefits to establish an effective feeding mechanism for children with CLP.

A limitation of the study was the small number of study participants. However, we are convinced that we were able to collect adequate data from a few excellent specialists and that this has enabled us to reveal all aspects of the Kumagai method.

Additionally, we limited the study participants to nurses. By recruiting other professionals, other techniques might have been discovered. However, contradictory statements could have been obtained from different professionals [15]. The Kumagai method is an established technique that has been trusted by oral surgeons, pediatricians, and feeding and swallowing specialists since its development by Ms. Kumagai, and families who have been taught the Kumagai method have been able to establish successful feeding routines for children with CLP, which is a testament to the method’s reproducibility.

### 4.2. Further Research

In the present study, we revealed the methods developed by specialists based on their daily experiences. Only one case report showed that feeding speed was improved from 40 mL/60 min to 40 mL/10 min using the Kumagai method in children with CLP [22]. However, evidence of this method’s efficacy was not obtained. Outcomes showing the effectiveness of feeding may include an improved sucking ability, less spillage, less coughing, increased volume intake, improved weight gain, and less fatigue during and after feeding [27]. Further research will be required to clarify the extent to which these factors are improved when using the Kumagai method, for example, a randomized controlled trial comparing the Kumagai method using the Long Nipple against standard care.

During our interviews with participants and the subsequent data collection, the techniques we gathered were limited to those the participants were consciously aware of. They may also be utilizing unconscious or implicit methods. Therefore, observing situations where participants are instructing parents on how to feed their children with CLP may be beneficial, as this could lead to the discovery of previously missed nuances about the optimal technique.

Furthermore, it is also important to address the child’s response, as the actual child may resist the Long Nipple or refuse to suckle. Further research should aim to clarify the experts’ strategies with to overcome issues of real children who struggle to accept the standardized technique.

### 4.3. Implications for Policy and Practice

The main nutrition providers for children are their parents, who should fully master the special technique of the Long Nipple. Currently, only a few experts teach this technique directly to parents, most of whom have been able to master the method. The results of this study may allow for a more systematic and streamlined educational approach for this technique with consequently more efficient learning, even if the teaching does not come directly from an expert.

The Long Nipple is still rarely used in clinical practice. This study may lead to a more widespread use of Long Nipples and improve the nutritional status of infants with CLP who have feeding difficulties, if the proper procedures are followed throughout the feeding process. Certainly, there is still room for verification, as noted in Section 4.2 “Further Research”. However, the participating specialists have already realized the effects of the Kumagai method for many children with feeding difficulties, including children with Robin sequence. Although verification of this positive impact is necessary, the related empirical knowledge must be made available to others for widespread clinical practice. Patient outcome data of rare diseases such as Robin sequence are lacking worldwide. Recently, a multicenter and multinational registration has been conducted and would assess the outcomes of different treatment approaches [28]. With the spread of the Kumagai method and the utilization of the findings of this registry, various benefits will become apparent in the future.

The written description of these procedures alone may not suffice to interiorize all movements and aspects involved in the technique to put it into practice effectively. To master the Kumagai method, receiving direct instructions from specialists may be necessary. In recent years, given their effectiveness, learning tools utilizing virtual reality for acquiring nursing skills have been suggested [29,30]. Our goal is to create a society where individuals who require the Kumagai method can develop the necessary skills through technology. This may involve using virtual reality avatars, which can demonstrate the correct feeding movements while displaying synchronization rates and providing step-by-step explanations to facilitate proper imitation of the correct movements. Through these technologies, individuals can acquire the skills required to implement the Kumagai method with greater ease and accuracy in the future.

## 5. Conclusions

We analyzed and described the elements of the Kumagai method, a procedure for using a Long Nipple for children with CLP. The method comprises several specific techniques: “cuddling as if pulling the child toward oneself” and “holding the child straight in a slightly upright position” as the basic posture for feeding, “holding the bottle with three fingers” and “pinching the center of the nipple between two fingers” as bottle holding, “moving the bottle horizontally and lightly placing the nipple against the lower lip”, “placing the nipple into the mouth so that it adheres to the lower lip and the tongue”, and “pushing milk out by pinching the nipple between two fingers” as nipple insertion, “pinching the nipple in time with the sucking” and “adjusting the pinching force according to the child’s sucking ability” as maintaining feeding, and “pulling the nipple straight out” as the end of feeding. Each of these techniques involves multiple steps. These items were found to be steps toward achieving the goals of the Kumagai method, namely, “encouraging self-sucking with a closed mouth” and “being chokubo-zui (in children without a cleft palate)”.

## Figures and Tables

**Table 1:** Differences in feeding tools commonly used in children with CLP.

	General Bottle	PCPSB, Medela SNF	Syringe, Spoon, Cup	Long Nipple
Method to facilitate milk extraction	The nipple is placed in the child’s mouth letting the child suckle	Bottle or nipple squeezing	The milk is poured into the child’s mouth	Unknown
Need for the child to create negative pressure for the milk to flow out	Yes	No	No	No
Nipple shape characteristics	Various sizes	Somewhat thick	No nipple	Thin and long
Cost	Varying	Expensive	Cheap	Cheap

PCBPB: Pigeon Cleft Palate Soft Bottle, Medela SNF: Medela Special Needs Feeder.

**Table 2:** Feeding preparation. (**a**) Basic Posture; (**b**) How to Hold the Bottle.

**(a)**	
**Category**	**Subcategory**
Cuddling as if pulling the child toward oneself.	Place the child’s nuchal region closely on the inside of the elbow joint and pull the child close to your breast.
	Restrict the child’s movement by holding the child’s arm under the armpit and by keeping the child’s body close to your body.
	Support the child’s body by holding it at the border between the buttocks and thighs.
	When feeding while sitting down, rest the child’s buttocks on your thighs.
Holding the child straight in a slightly upright position.	Hold the child in a slightly upright position.
	Hold the child’s body straight so that the head is not excessively bent forward or backward.
**(b)**	
**Category**	**Subcategory**
Holding the bottle with three fingers.	Ensure that the bottle holder is down.
	Ensure that the slit of the nipple tip is not directly below the bottle.
	Hold the bottle by the thumb, ring finger, and pinky finger.
Pinching the center of the nipple between the index and middle fingers.	Pinch the nipple between the terminal joints of the index and middle fingers.
	Pinch the nipple near the center of the thicker part of the nipple.
	Slightly bend and relax the pinching fingers.

**Table 3:** Example of the coding and categorizing process (excerpt of the raw data concerning “Basic posture”).

Raw Data (Participant D)	Codes	Subcategories
I tuck the child’s arm under my armpit (1). That helps to keep the upper limbs in place (2) and the child’s body in close contact with my body (3). The arm is always tucked under the armpit. My hand provides support at the border between the child’s thigh and buttock (4). The important point is to make sure that the child’s nuchal region can be held in close contact with the V-shaped part (the inside) of the elbow joint (5).	(1) Tucking the child’s arm under the armpit. (2) Keeping the upper limbs in place. (3) Keeping the child’s body in close contact with the carer’s body. (4) Supporting the child’s body by holding the border between the child’s thigh and buttock (5) Holding the child’s nuchal region in close contact with the V-shaped part of the inner elbow joint.	Restrict the child’s movement by holding the child’s arm under the armpit and by keeping the child’s body close to your body. (Codes of (1) and (3) were included in this) Place the child’s nuchal region closely on the inside of the elbow joint and pull the child close to your breast. (The codes of (2) and (5) were included in this) Support the child’s body by holding it at the border between the buttocks and thighs. (The code of (4) was included in this)

Underlining means each segment.

**Table 4:** Feeding procedure. (**a**) Nipple Insertion into the Mouth; (**b**) Maintaining Feeding; (**c**) End of Feeding.

**(a)**	
**Category**	**Subcategory**
Moving the bottle horizontally and lightly placing the nipple against the lower lip.	Move the bottle horizontally and lightly place the nipple against the lower lip.
Placing the nipple into the mouth so that it adheres to the lower lip and the tongue.	Insert the nipple into the mouth when the mouth is open.
	Smoothly place the nipple in the mouth so that it adheres to the lower lip and the tongue.
	Control the angle of insertion with the wrist.
	Place the nipple on the midline and center of the tongue.
	Tilt the nipple (in contact with the tongue surface) about 10 degrees so that the milk fills it.
	Insert the nipple into the mouth up to 2–3 mm from the start of the thicker segment of the nipple, just touching the child’s lip (just point).
	When moving the bottle, relax your arm, keep your side lightly tight, and do not raise your elbow.
	Keep the nipple straight at all times.
Pushing the milk out by pinching the nipple between the index and middle fingers.	Pinch the nipple when milk fills it.
	Pinch the nipple, applying equal force with the index and middle fingers.
	The time from placing the nipple on the lower lip and pinching the nipple should be approximately 2 s.
**(b)**	
**Category**	**Subcategory**
Pinching the nipple in time with the sucking.	Pinch the nipple when the child is sucking with the mouth closed.
	Do not pinch during child inspiration.
	Keep your fingers on the nipples even when you are not pinching.
Adjusting the pinching force according to the child’s sucking ability.	Pinch with a force that will allow for a 1–2 cm flow of air bubbles into the nipple when sucking.
	Feed the target amount of milk at a pace of approximately 15 min.
**(c)**	
**Category**	**Subcategory**
Pulling the nipple straight out.	Pull the nipple straight out at the angle of the oral cavity.
	If milk remains in the nipple, turn the nipple up immediately after pulling it out.

## Data Availability

The data presented in this study are available on request from the corresponding author. The data are not publicly available due to restriction for ethical consideration to each participant.
